# The relationship between sleep onset time and cardiometabolic biomarkers in Chinese communities: a cross-sectional study

**DOI:** 10.1186/s12889-020-08516-9

**Published:** 2020-03-20

**Authors:** Liqun Wang, Jiangping Li, Yong Du, Ting Sun, Li Na, Zhizhong Wang

**Affiliations:** 1 School of Public Health and Management, Ningxia Medical University, Yinchuan, 750004 China; 2 Surgical Laboratory of General Hospital, Ningxia Medical University, Yinchuan, 750004 China; 3 School of Clinical Medicine, Ningxia Medical University, Yinchuan, 750004 China

**Keywords:** Sleep onset time, Obesity, Triglyceride, High-density lipoprotein, Fasting plasma glucose, Cross-sectional study

## Abstract

**Background:**

Late sleep onset time (SOT) is a common social phenomenon in modern society, and it was associated with a higher risk of obesity. However, the literature gap exists about the SOT and cardiometabolic biomarkers which closely associated with obesity. The present study aimed to explore the association of SOT with cardiometabolic biomarkers in Chinese communities.

**Methods:**

A cross-sectional study enrolled a total of 2418 participants was conducted in Ningxia province of China. The cardiometabolic biomarkers included triglyceride, total cholesterol, high-density lipoprotein, low-density lipoprotein and fasting plasma glucose were measured quantitatively using the standard method. The SOT and sleep duration were acquired by a self-report questionnaire. The multiple mixed-effect linear regression model was employed to examine the association.

**Results:**

Binary analysis found an inverse association of SOT with high-density lipoprotein (β = − 0.05, 95%*CI*: − 0.06, − 0.03), with 1 h delayed in SOT the high-density lipoprotein decreased 0.05 mmol/L. After controlling for demographic variables, health-related behaviors, and physical health covariates, late SOT was associated with a higher level of triglyceride (β = 0.12, 95%*CI*: 0.06, 0.18), a higher level of low-density lipoprotein (β = 0.06, 95% *CI*: 0.02, 0.09), and a lower level of high-density lipoprotein (β = − 0.05, 95% *CI*: − 0.06, − 0.03). when stratified by sleep duration (less than eight hours vs. eight and longer hours), a positive association between SOT and LDL (β = 0.08, 95% *CI*: 0.04, 0.12) was found among participants with sleep duration eight hours and longer.

**Conclusion:**

Late sleep onset time with the negative effect on the cardiometabolic biomarkers, and individuals with late SOT coupled with longer sleep duration may take risk of a higher level of low-density lipoprotein which in turn lead to increase the risk of cardiovascular disease.

## Background

Sleep is an important psychological and biochemical phenomenon in human beings, the quality of sleep is a basic measure of health status [1]. High quality sleep played an important role in maintaining physical health as well as mental health [2]. Appropriate sleep can help eliminate fatigue, accumulate energy, repair damage, and regulate the function of the body [3]. Conversely, sleep deprivation or short sleep duration could lead to adverse health conditions [4], endocrine disorders [5], memory decline [6], depression [7], cardiovascular disease [8], and total mortality [9]. Studies have reported that short sleep duration associated with an increased risk of obesity [10–13].

Sleep onset time (SOT) or bedtime is one of the sleep patterns and is important for the rhythm of sleeping at an early lifetime. Late SOT had a significant influence on energy intake and expenditure. Many studies have reported that late SOT associated with a higher risk of obesity [14–17]. Besides, a study has found that early bedtime associated with a higher quality of dietary and compliance rate of physical activity guidelines [18]. Late SOT usually results in short sleep duration [19], studies have found that short sleep duration associated with weight gain via hormonal responses involving reciprocal changes in leptin and ghrelin levels, leading to reduced energy expenditure and increased appetite and energy intake [20, 21].

However, the literature gap still exists about the SOT and cardiometabolic biomarkers which have a close relationship with obesity. Plasma lipids is one of the most sensitive biochemical markers of obesity [22], the level of triglyceride (TG), total cholesterol (TC), low-density lipoprotein (LDL) strongly positively correlated with body mass index (BMI), and the level of high-density lipoprotein (HDL) negatively correlated with BMI [23, 24]. And the studies have suggested that obesity would be associated with a higher level of fasting plasma glucose (FPG) [25–27]. While studies have found late SOT associated with a higher degree of insulin resistance in midlife women [28] and with a higher level of fasting glucose in adults with diabetes [29]. There no study directly linked SOT with those cardiometabolic biomarkers which are proximate causes of cardiovascular disease.

Hence, the current study aimed to explore the relationship between SOT and cardiometabolic biomarkers among a random Chinese community population. We hypothesized that late SOT was associated with higher levels of TG, TC, LDL or FPG, and lower level of HDL in general community adults.

## Method

### Study design

Cross-sectional study design conducted from April to June 2017.

### Participants

The data of this study abstracted from the project of Ningxia Hui Autonomous Region 13th Five-Year Technology Major Project, which was aimed to exploring the clinical function and application of intestinal flora in chronic diseases. A multi-stage sampling protocol was used to select the subjects in the project. In summary, first, four counties/ districts (Xingqing District, Litong District, Pengyang County, and Xiji County) were selected from a total of 22 counties/districts using the stratified sampling design. The target counties/districts were classified into four stratus depending on the proportion of the minority population and the economic status (less minority population with lower economic status, less minority population with higher economic status, more minority population with lower economic status, more minority population with higher economic status). Second, ten communities were selected from each district/county using random sampling stratified by urban and rural areas, results a total of forty communities consist of twenty rural communities and twenty urban communities forward to the next step. Third, 115 households were selected in each community by the systematic sampling method. Finally, the KISH Table was used to select one eligible family member from each household, there were 615 households do not get touched after three times attempts which result in a total of 3985 eligible participants were selected and be invited to receive a face-to-face survey and physical examination. Of them, 2418 participants accepted the invitation and finished both the full questionnaire and physical examination with cardiometabolic biomarkers test include in the final data analysis. The response rate in the rural area slightly higher than in the urban community (65.5% vs. 60.8%).

The inclusion criteria for this study were as follows: a) living at the present address for least 6 months, and; b) age ranging from 18 to 80 y. The exclusion criteria were as follows: a) unconsciousness caused by any forms condition; b) the acute phase of a cerebrovascular accident; c) a severe illness that prevents communication; d) any obvious cognitive disabilities or deafness, aphasia or other language barriers; and e) with sleep disorders and taking hypnotics, as well as some particular work need to going to bed late.

### Measures

#### Dependent variables

All the participants underwent a careful physical examination and provide the blood sample for cardiometabolic biomarkers test. All those laboratory tests were finished in the hospital laboratory according to standard procedure.

#### Independent variables

A face-to-face interview was performed by trained medical students using a structured questionnaire. One item question “What time do you usually go to sleep at night?”, another item question “What time do you usually rise in the morning?” asked to identify SOT and sleep duration. The SOT was divided into six groups as before 8:00 pm, 8:00 pm-9:00 pm, 9:00 pm-10:00 pm, 10:00 pm-11:00 pm, 11:00 pm-12:00 midnight, and after 12:00 midnight.

#### Health-related behaviors

Health-related behaviors included smoking, alcohol use, tea-drinking, exercise. Those health-related behavior variables employed in this paper were operationally defined. Smoking was defined as at least one cigarette per day and last for 6 months or more. Alcohol use was defined as at least one glass of alcohol use in the past 12 months. Tea drinking frequency was assessed by asking the question “How often do you drink tea (days per week)?” with the possible response: once a day or more, 5–6 times/week, 3–4 times/week, 1–2 times/week, less than once a week and never. Physical exercise was assessed by asking the question “Do you perform at least 30 minutes of physical activity at work and/or leisure time more than 4 days a week?”, with a yes/ no response.

#### Physical health

Physical health characteristics include diabetes (yes vs. no) and hypertension (yes vs. no). The diabetes mellitus (DM) was diagnosed according to the WHO criteria through performing oral glucose tolerance test (OGTT) when a two hours post glucose load over 11.1 mmol/L was defined as DM. Hypertension was defined as systolic blood pressure ≥ 140 mmHg or diastolic blood pressure ≥ 90 mmHg. Weight and height were measured by trained nurses according to standard instruction, body mass index (BMI) was calculated with the formula: BMI = weight (kg)/height (m)^2^.

#### Demographic variables

Demographic information collected included age, gender, education (was measured with question: how many years of school education do you have"), marital status (married vs. unmarried), residence (rural vs. urban), ethnicity (Han vs. minority), occupation, family income (measured by the self-reported family average individual income per month (in local currency RMB) and was divided into five groups: < 1000, 1000-1999, 2000-2999, 3000–4999 and 5000 or more).

### Statistical analyses

All the analysis was performed using the Software for Statistics and Data Science (STATA) 14.0. The quantitative variables were described as means (median) and standard deviations (quartiles). Categorical variables were described as counts and proportions. Differences in demographic, health-related behaviors and psychical health between urban participants and rural participants were examined using the Student’s t-test for quantitative variables and the chi-square test for categorical variables. The cardiometabolic biomarkers were examined using the Wilcoxon rank-sum test. The multiple mixed-effect linear regression was employed to examine the association between SOT and the cardiometabolic biomarkers, three separate models were launched to control the covariates step by step, in model 1 adjusted for demographic variables (age, gender, ethnicity, education, marital status, occupation, economic condition); The model 2 based on model 1 plus health-related behaviors (smoking, alcohol use, tea-drinking, physical exercise); then the model 3 based on model 2 plus physical health (diabetes mellitus, hypertension, BMI). Of the independent variables, the rural/urban was fitted as a random intercept model. Due to the possible interaction of SOT and sleep duration, we performed the regression process stratified by sleep duration for models 1 to 3. The results of the regression models are summarized via beta coefficients (slopes for continuous measures, the beta coefficients reported for SOT refer to every one hour delay in SOT, and differences between groups for categorical measures) and their 95% confidence intervals.

## Results

### Demographic characteristics of the participants

As shown in Table 1, the total sample has an average age of 52.5 (SD = 12.9) years, with a range of 18 to 80 years. Slightly more than half (63.0%) were female, and 48.7% were farmers, with an average educational year of 5.8 (SD = 4.7) years. The mean sleep duration was 8.1(SD = 1.3) hours, 42.9% of the participants go to bed at 10:00 pm-11:00 pm, and more than a quarter of them at 11:00 pm-12:00 midnight.

**Table 1:** The demographic characteristics of the participants

Variables	Total *n* = 2418	Urban *n* = 1150	Rural^a^ *n* = 1268	*P* value
Age, mean (SD), years	52.5 (12.9)	52.4 (13.1)	52.6 (12.8)	0.739
Gender, n (%)				0.003
female	1523 (62.99)	760 (66.09)	763 (60.17)	
male	895 (37.01)	390 (33.91)	505 (39.83)	
Ethnicity, n (%)				< 0.001
Han	1516 (62.70)	878 (76.35)	638 (50.32)	
minority	902 (37.30)	272 (23.65)	630 (49.68)	
Marital status, n (%)				0.359
married	2188 (90.49)	1034 (89.91)	1154 (91.01)	
unmarried	230 (9.51)	116 (10.09)	114 (8.99)	
Occupation, farmer, n (%)	1177 (48.70)	231 (19.60)	946 (80.37)	< 0.001
Education, years, mean (SD)	5.8 (4.70)	7.6 (4.90)	4.2 (3.90)	< 0.001
FCMI, n (%)				< 0.001
< 1000	1032 (42.68)	303 (26.35)	729 (57.49)	
> 1000	1386 (57.32)	847 (73.65)	539 (42.51)	
Smoking, n (%)				0.221
Yes	419 (17.33)	186 (16.17)	233 (18.38)	
No	1905 (78.78)	914 (79.48)	991 (78.15)	
Once smoking	94 (3.89)	50 (4.35)	44 (3.47)	
Alcohol use, n (%)				0.015
Yes	401 (16.58)	213 (18.52)	188 (14.83)	
No	2017 (83.42)	937 (81.48)	1080 (85.17)	
Drinking tea, n (%)				< 0.001
Every day	965 (39.99)	371 (32.37)	594 (46.88)	
5–6 days a week	29 (1.20)	14 (1.22)	15 (1.18)	
3–4 days a week	66 (2.74)	31 (2.71)	35 (2.76)	
1–2 days a week	50 (2.07)	21 (1.83)	29 (2.29)	
Occasion	497 (20.60)	274 (23.91)	223 (17.60)	
Never	806 (33.40)	435 (37.96)	371 (29.28)	
Physical exercise, n (%)				< 0.001
Yes	2041 (85.58)	919 (81.11)	1122 (89.62)	
No	374 (14.42)	214 (18.89)	162 (10.38)	
Hypertension, n (%)				0.077
Yes	532 (22.00)	271 (23.57)	261 (20.58)	
No	1886 (78.00)	879 (76.43)	1007 (79.42)	
Diabetes mellitus, n (%)				0.017
Yes	128 (5.29)	74 (6.43)	54 (4.26)	
No	2290 (94.71)	1076 (93.57)	1214 (95.74)	
BMI, mean (SD), kg/m^2^	25.4 (8.5)	25.0 (3.8)	25.7 (11.3)	0.041
Sleep duration, mean (SD), hour	8.1 (1.3)	8.0 (1.2)	8.1 (1.4)	0.002
Sleep onset time, n (%)				< 0.001
Before 8:00 pm	19 (0.78)	5 (0.43)	14 (1.10)	
8:00 pm-9:00 pm	145 (6.00)	35 (3.04)	110 (8.68)	
9:00 pm-10:00 pm	503 (20.80)	170 (14.78)	333 (26.26)	
10:00 pm-11:00 pm	1036 (42.85)	466 (40.52)	570 (44.95)	
11:00 pm-12:00midnight	685 (28.33)	461 (40.09)	224 (17.67)	
After 12:00midnight	30 (1.24)	13 (1.13)	17 (1.34)	
Triglycerides, M (Q), mmol/L	1.2 (0.8,1.9)	1.2 (1.2,1.9)	1.2 (0.8,1.8)	0.192
Total cholesterol, M (Q), mmol/L	3.6 (3.1,4.2)	3.6 (3.1,4.1)	3.7 (3.2,4.3)	< 0.001
HDL, median (Q), mmol/L	1.1 (0.9,1.2)	1.1 (0.9,1.3)	1.1 (0.9,1.3)	0.056
LDL, median (Q), mmol/L	2.5 (2.1,3.0)	2.6 (2.6,3.0)	2.5 (2.0,3.0)	0.006
FPG, median (Q), mmol/L	4.5 (4.1,5.0)	4.6 (4.3,5.1)	4.4 (4.1,4.9)	< 0.001

^a^: compared with urban; *SD* Standard deviation, *Q* Quartile, *FCMI* Family per capita monthly income, *HDL* High-density lipoprotein cholesterol, *LDL* low-density lipoprotein cholesterol, *FPG* fasting plasma glucose;

The participants living in the rural area had lower educational attainment, lower alcohol use prevalence and more physical exercise than those living in the urban area (*P* < 0.05). In addition, participants living in the rural area had longer sleep duration, earlier SOT, higher level of TC, lower level of LDL and lower level of FPG than those living in the urban area.

### Bivariate regression model

**Fig. 1:**
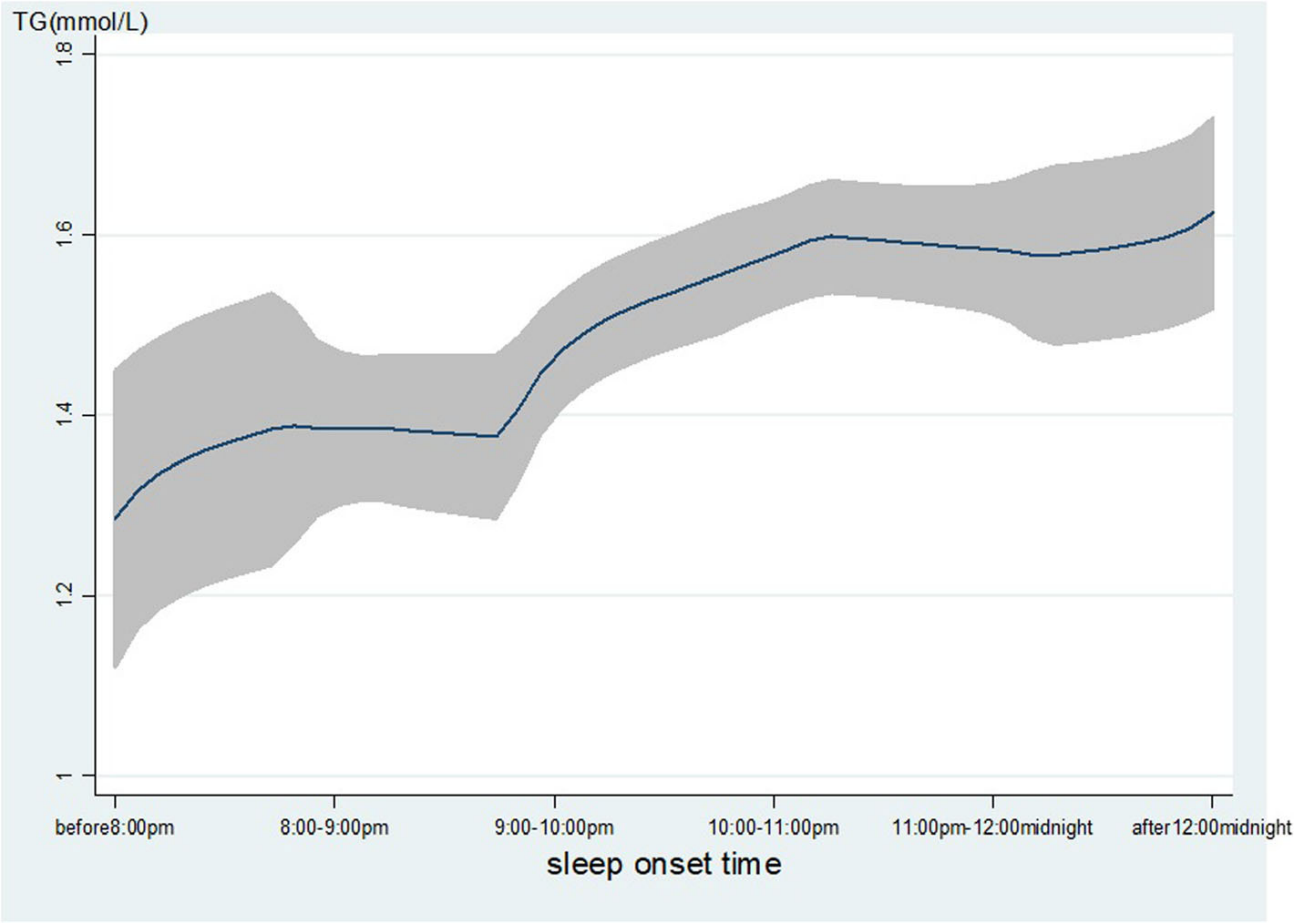
The level of TG with the change of SOT. The level of TG (mmol/L) was increased with the delay of sleep onset time. The line represents the value of TG, the gray confidence intervals represent the 95% confidence intervals of TG

**Fig. 2:**
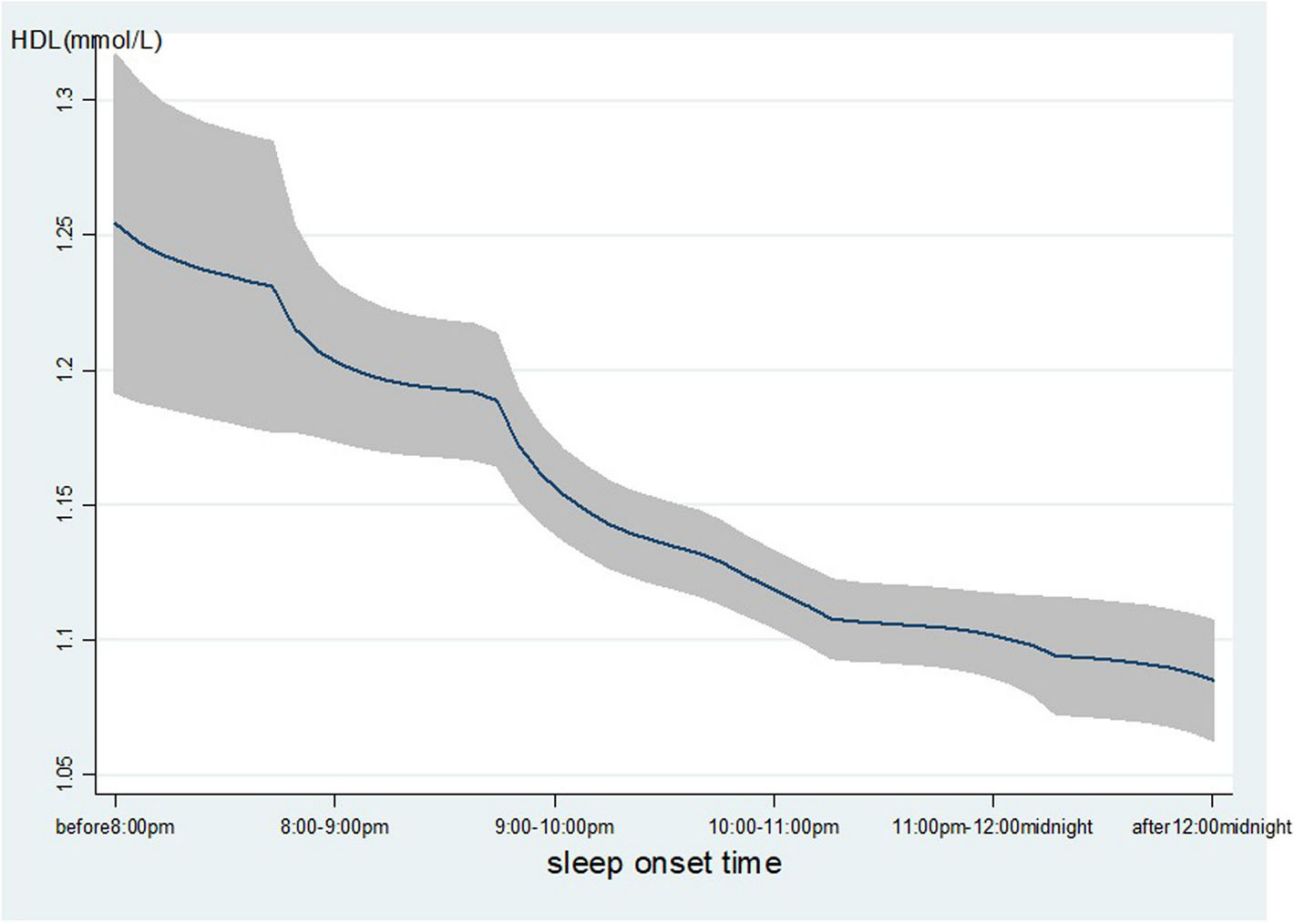
The level of HDL with the change of SOT. The level of TG (mmol/L) was decreased with the delay of sleep onset time. The line represents the value of HDL; the gray confidence intervals represent the 95% confidence intervals of HDL

As shown in Table 2, age, gender, ethnicity, occupation, family income, smoking, alcohol use, hypertension, DM and BMI were associated with the level of TG. Gender, ethnicity, marital status, occupation, education, family income, smoking, alcohol use, tea-drinking, hypertension, DM and BMI were associated with the level of HDL. The late SOT was associated with lower level of HDL (β = − 0.05, 95% *CI*: − 0.06, − 0.03), that means with 1-h delay in SOT will decrease the high-density lipoprotein 0.05 mmol/L, while the sleep duration was positively associated with HDL (β = 0.02, 95% *CI*: 0.01, 0.03). Furthermore, late SOT was associated with a higher level of TG (β = 0.10, 95% *CI*: 0.04, 0.15). As displayed in Fig. 1**,** with the delay of SOT, the level of TG increased significantly. In contrast, with the delay of SOT, the level of HDL decreased significantly as Fig. 2 displayed.

**Table 2:** Bivariate regression model (*n* = 2418)

	Triglycerides *β* (95%*CI*)	Total cholesterol *β* (95%*CI*)	HDL *β* (95%*CI*)	LDL *β* (95%*CI*)	FPG *β* (95%*CI*)
Age	0.01 (0.00,0.014)**	−0.11(− 0.36,0.14)	0.00(− 0.00,0.00)	0.01 (0.01,0.02)**	0.00(− 0.00,0.00)
Gender	− 0.15(− 0.26,-0.05)**	−4.39(− 11.1,2.35)	0.13 (0.10,0.15)**	− 0.00(− 0.06,0.06)	0.03 (0.00,0.06)*
Ethnicity	0.10 (0.00,0.21)*	4.40(− 2.33,11.13)	− 0.08(− 0.10,-0.05)**	− 0.04(− 0.10,0.01)	−0.03(− 0.06,0.00)
Martial status	− 0.02(− 0.19,0.14)	−0.79(− 11.5,9.95)	0.06 (0.02,0.10)**	0.16 (0.07,0.26)**	0.02(− 0.03,0.07)
Occupation	0.02 (0.01,0.04)**	0.53(−0.32,1.38)	−0.00(− 0.01,-0.00)*	0.01 (0.00,0.02)**	0.00(− 0.00,0.00)
Education	0.00(− 0.01,0.01)	−0.07(− 0.75,0.61)	−0.01(− 0.01,− 0.00)**	-0.00(− 0.01,0.00)	0.00(− 0.00,0.00)
FCMI	0.07 (0.03,0.12)**	−1.27(− 4.00,1.45)	−0.03(− 0.04,-0.02)**	0.01(− 0.01,0.03)	0.01(− 0.01,0.02)
Smoking	−0.17(− 0.28,− 0.06)**	9.75 (2.34,17.1)*	0.08 (0.05,0.10)**	-0.06(− 0.12,0.01)	0.03(− 0.01,0.06)
Alcohol use	−0.17(− 0.31,-0.04)*	1.97(−6.79,10.72)	0.06 (0.02,0.09)**	−0.03(− 0.10,0.05)	0.02(− 0.03,0.06)
Tea drinking	0.00(− 0.02,0.02)	−0.88(− 2.32,0.57)	0.01 (0.01,0.02)**	− 0.02(− 0.03,-0.01)**	0.01 (0.00,0.01)*
Physical exercise	0.13(− 0.01,0.27)	−2.02(− 11.4,7.34)	−0.01(− 0.05,0.02)	−0.00(− 0.08,0.08)	−0.01(− 0.05,0.03)
Hypertension	− 0.42(− 0.54,-0.30)**	1.90(−5.97,9.78)	0.07 (0.04,0.10)**	−0.20(− 0.27,-0.13)**	0.05 (0.01,0.09)**
Diabetes mellitus	−0.73(− 0.95,-0.51)**	0.51(− 13.0,16.1)	0.14 (0.08,0.19)**	−0.19(− 0.32,-0.06)**	0.50 (0.44,0.57)**
Body mass index	0.02 (0.01,0.03)**	0.01(−0.37,0.39)	−0.002(− 0.004,-0.001)**	0.006 (0.002,0.009)**	0.00(− 0.00,0.00)
Sleep duration	− 0.07(− 0.11,− 0.03)**	0.86(− 1.62,3.34)	0.02 (0.01,0.03)**	-0.03(− 0.06,-0.01)**	0.00(− 0.01,0.01)
Sleep onset time	0.10 (0.04,0.15)**	0.07(−3.46,3.61)	−0.05(− 0.06,-0.03)**	0.00(− 0.03,0.03)	−0.01(− 0.02,0.01)

**:*P* < 0.01; *p < 0.05; *β*: beta coefficient; *95%CI*: 95% confident interval, *FCMI* Family per capita monthly income;

*HDL* High-density lipoprotein cholesterol, *LDL* low-density lipoprotein cholesterol, *FPG* fasting plasma glucose;

### Multiple mixed-effect linear regression model between sleep onset time and cardiometabolic biomarkers

As showed in Table 3, in model 1, an inverse association between SOT and HDL (β = − 0.05, 95% *CI*: − 0.06, − 0.03), and a positive association between SOT and TG (β = 0.12, 95% *CI*: 0.06, 0.18), LDL (β = 0.06, 95% *CI*: 0.02, 0.09) were found after controlling for the demographic variables. The association persisted when controlling for health-related behaviors variables (Model 2), and psychical health variables (Model 3). No significant association was found between SOT and TC or FPG among any of the three models.

**Table 3:** Multiple mixed-effect linear regression models between sleep onset time and cardiometabolic biomarkers (*n* = 2418)

Models	Triglycerides *β* (95%*CI*)	Total cholesterol *β* (95%*CI*)	HDL *β* (95%*CI*)	LDL *β* (95%*CI*)	FPG# *β* (95%*CI*)
Model 1	0.12 (0.06,0.18)**	−0.50(−4.33,3.33)	−0.05(− 0.06,-0.03)**	0.06 (0.03,0.09)**	−0.02(− 0.03,0.01)
Model 2	0.12 (0.06,0.18)**	−0.76(− 4.62,3.10)	−0.05(− 0.06,-0.03)**	0.05 (0.02,0.09)**	−0.01(− 0.03,0.01)
Model 3	0.12 (0.06,0.18)**	−0.75(− 4.63,3.12)	−0.05(− 0.06,-0.03)**	0.06 (0.02,0.09)**	−0.01(− 0.03,0.01)

Model 1: sleep onset time + demographic characteristics (age, gender, ethnicity, education, marital status, occupation, economic condition); Model 2: Model 1 + health-related behaviors (smoking, alcohol use, tea drinking, physical exercise); Model 3: Model 2 + physical health (diabetes mellitus, hypertension, BMI); # The model does not include diabetes mellitus; **:*P* < 0.01; *β*: *β*: beta coefficient; *95%CI*: 95% confident interval; *HDL* High-density lipoprotein cholesterol, *LDL* low-density lipoprotein cholesterol, *FPG* fasting plasma glucose, All the β are for sleep onset time

There are about 2% of participants have bedtimes before 8 pm or after 12 midnight, sensitive tests conducted by excluding those participants with a bedtime before 8 pm or after 12 midnight (As shown in Supplementary Table 1). When the model stratified by sex, a positive association between SOT and TG (β = 0.17, *p* < 0.01), as well as LDL (β = 0.09, *p* < 0.01) was found among males instead of females (showed in Supplementary Table 2).

### Multiple mixed-effect linear regression model between sleep onset time and LDL stratified by sleep duration

As shown in Table 4, when stratified by sleep duration (less than eight hours vs. eight and longer hours). A positive association between SOT and LDL (β = 0.08, 95% *CI*: 0.04,0.12) was found among participants with sleep duration eight hours and longer.

**Table 4:** Multiple mixed-effect linear regression models between sleep onset time and cardiometabolic biomarkers stratified by sleep duration

Less than 8 h (*n* = 837)					
	Triglycerides *β* (95%*CI*)	Total cholesterol *β* (95%*CI*)	HDL *β* (95%*CI*)	LDL *β* (95%*CI*)	FPG# *β* (95%*CI*)
Model 1	0.09(−0.04,0.22)	− 0.02(− 0.10,0.06)	−0.04(− 0.07,-0.01)**	−0.02(− 0.09,0.05)	−0.003(− 0.04,0.04)
Model 2	0.07(− 0.06,0.20)	−0.03(− 0.11,0.05)	−0.04(− 0.07,-0.01)*	−0.03(− 0.10,0.04)	−0.001(− 0.04,0.04)
Model 3	0.08(− 0.04,0.21)	−0.03(− 0.12,0.05)	−0.04(− 0.07,-0.01)**	−0.03(− 0.10,0.04)	−0.002(− 0.04,0.04)
8 h and longer (*n* = 1581)					
	Triglycerides *β* (95%*CI*)	Total cholesterol *β* (95%*CI*)	HDL *β* (95%*CI*)	LDL *β* (95%*CI*)	FPG# *β* (95%*CI*)
Model 1	0.16 (0.09,0.24)**	0.10(−6.21,6.41)	−0.06(− 0.08,-0.04)**	0.10 (0.05,0.14)**	− 0.02(− 0.04,0.01)
Model 2	0.15 (0.07,0.23)**	0.55(−5.91,7.02)	− 0.06(− 0.08,-0.04)**	0.09 (0.04,0.13)**	−0.02(− 0.04,0.01)
Model 3	0.13 (0.05,0.20)**	0.48(− 5.99,6.95)	− 0.05(− 0.07,-0.03)**	0.08 (0.04,0.12) **	−0.02(− 0.04,0.01)

Model 1: sleep onset time + demographic characteristics (age, gender, ethnicity, education, marital status, occupation, economic condition); Model 2: Model 1 + health-related behaviors (smoking, alcohol use, tea drinking, physical exercise); Model 3: Model 2 + physical health (diabetes mellitus, hypertension, BMI)

# The model does not include diabetes mellitus; ** *p* < 0.01 *p < 0.05; *β*: beta coefficient; *95%CI*: 95% confident interval, *HDL* High-density lipoprotein cholesterol, *LDL* low-density lipoprotein cholesterol, *FPG* fasting plasma glucose, All the β are for sleep onset time

## Discussion

To our knowledge, this is one of the first studies focused on the association of SOT with cardiometabolic biomarkers among a random Chinese sample. The results revealed that late SOT associated with a lower level of HDL, a higher level of TG as well as LDL, after controlling for the potential confounders using the mixed-effect linear regression model. The findings indicate that individuals going to bed late at night (example 11:00 pm or later) had a higher level of bad lipids (TG, LDL) and a lower level of good lipids (HDL), consequently, may lead to increased risk of cardiovascular disease. The findings consistent with further data as showed in the Supplementary Figures 1, 2 and 3, according to the cut point for metabolic risk suggested by Chinese Guidelines on Prevention and Treatment of Dyslipidemia in Adults (2007), the abnormal prevalence of HDL for participants with SOT of before 20:00 pm, 8:00–9:00 pm, 9:00–10:00 pm, 10:00–11:00 pm, and 11:00 pm-12:00midnight were 21.1, 33.8, 34.6, 46, 47.3, 66.7%, respectively; the abnormal prevalence of TG was 5.3, 26.2, 25.4, 32.3, 32.1, 53.3%, respectively; and the abnormal rate of LDL was 5.3, 12.4, 11.5, 12.6, 12.1, 6.7%, respectively. Our findings were supported by the previous study that suggested late SOT associated with a higher risk of obesity (which was believed causes a lower level of HDL, and a higher level of LDL) [24]. Besides, a study has suggested SOT inversely correlated with sleep duration [21], and short sleep duration was associated with higher level of LDL and TG among adults [30]; Previous research also found an association between shorter sleep duration and higher risk of obesity (a determinants of cardiometabolic biomarkers) [31].

The stratified multivariate regression model in the present study also indicate possible interaction between SOT and sleep duration. The association between SOT and TG or LDL appeared among individuals with sleep duration eight hours and longer. No significant association was found between SOT and TG or LDL among individuals with a sleep duration less than eight hours. Theoretically, late bedtime coupled with longer sleep duration indicates a late bedtime-late rise sleep behavior, there at least one study has revealed that those individuals with a late bedtime and late rise sleep-wake behavior had a higher risk of weight gain, lower diet quality than any other sleep-wake behaviors in adolescents [32].

Our results displayed there was no association between SOT and FPG which inconsistent with existing literature. A study found that those who stay awake until late into the night are more likely to engage in smoking, late-night eating and a sedentary lifestyle, hence, associated with increased risk of type 2 diabetes [29]. Also, the previous study report that late bedtime significantly correlated with shorter sleep duration, as well as poorer glycemic control [19].

In addition, current study found that the average sleep duration was 8.1 h, which longer than the results reported among American adults on working days (less than 7 h per night) [33]. It may be due to the higher proportion of participants (52.6%) were living in the rural area where people with earlier SOT and longer sleep duration than in the urban area, which was consistent with the previous study [34]. Also, the participants were elder than the participants in the previous study may contribute to longer sleep duration.

### Limitations and strengths

The strength of our study is the population-based and multi-stage random sampling design, as well as a large sample size with blood biochemical indexes involved. However, this study has several limitations. First, given its cross-sectional design, causal relationships between SOT and cardiometabolic biomarkers cannot be determined. Second, bedtime and sleep duration were collected via a self-reported survey question; it may involve information bias despite it be commonly used in the epidemiological study due to the feasibility consideration. Third, the sample acquired from one of the provinces of China, thus, caution should be demonstrated when generalizing these findings to other areas of mainland China.

## Conclusion

Late sleep onset time was associated with a higher level of TG, and LDL, meanwhile, with a lower level of HDL. The associations were stronger in participants with longer sleep duration than those in participants with shorter sleep duration. The findings are helpful in explaining how late sleep onset time leads to an increased risk of obesity which in turn leads to increased risk of cardiovascular disease.

## Supplementary information

**Additional file 1: Figure S1.** Distribution of HDL among different SOT. The abnormal HDL was increased with the delayed SOT. The line parallel to the X axis was the cutoff reference line. The upper and lower edges of boxplot represent the upper and lower quartile and the short black line in boxplot represent the median. **Figure S2.** Distribution of TG among different SOT. The abnormal TG was increased with the delayed SOT. The line parallel to the X axis was the cutoff reference line. The upper and lower edges of boxplot represent the upper and lower quartile and the short black line in boxplot represent the median. **Figure S3.** Distribution of TG among different SOT. The line parallel to the X axis was the cutoff reference line. The upper and lower edges of boxplot represent the upper and lower quartile and the short black line in boxplot represent the median. **Table S1.** Regression model excludes participants with SOT bedtimes before 8:00 pm or after 12:00 midnight (*n* = 2369). **Table S2.** Regression models between sleep onset time and cardiometabolic biomarkers stratified by sex

## Data Availability

The dataset supporting the conclusions of this article is included in the article. Additional data are available upon individual request to Dr. Wang at wzhzh_lion@126.com.

## Abbreviations

SD: Standard deviation
BMI: Body mass index
SOT: Sleep onset time
TG: Triglyceride
TC: Total cholesterol
HDL: High density lipoprotein
LDL: Low density lipoprotein
FPG: Fasting plasma glucose
DM: Diabetes mellitus
