# Supplementary material for: The relationship between sleep onset time and cardiometabolic biomarkers in Chinese communities: a cross-sectional study
Source: BMC Public Health. 2020 Mar 20;20:374. doi: 10.1186/s12889-020-08516-9 (PMC7085179; doi:10.1186/s12889-020-08516-9)
Supplement: Supplementary file 1 — Additional file 1: Figure S1. Distribution of HDL among different SOT. The abnormal HDL was increased with the delayed SOT. The line parallel to the X axis was the cutoff reference line. The upper and lower edges of boxplot represent the upper and lower quartile and the short black line in boxplot represent the median. Figure S2. Distribution of TG among different SOT. The abnormal TG was increased with the delayed SOT. The line parallel to the X axis was the cutoff reference line. The upper and lower edges of boxplot represent the upper and lower quartile and the short black line in boxplot represent the median. Figure S3. Distribution of TG among different SOT. The line parallel to the X axis was the cutoff reference line. The upper and lower edges of boxplot represent the upper and lower quartile and the short black line in boxplot represent the median. Table S1. Regression model excludes participants with SOT bedtimes before 8:00 pm or after 12:00 midnight (n = 2369). Table S2. Regression models between sleep onset time and cardiometabolic biomarkers stratified by sex [file 12889_2020_8516_MOESM1_ESM.docx]

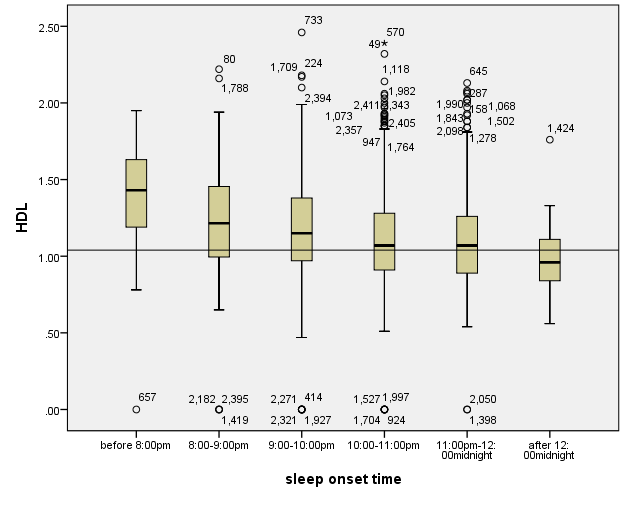


**Supplementary figure 1.** Distribution of HDL among different SOT. The abnormal HDL was increased with the delayed SOT. The line parallel to the X axis was the cutoff reference line. The upper and lower edges of boxplot represent the upper and lower quartile and the short black line in boxplot represent the median.


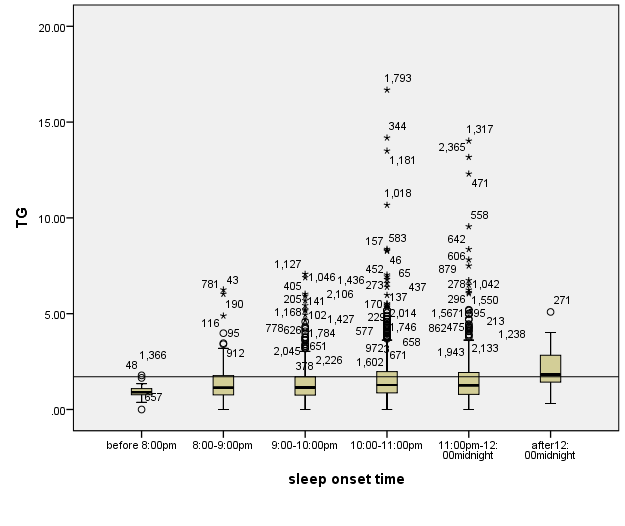


**Supplementary figure 2.** Distribution of TG among different SOT. The abnormal TG was increased with the delayed SOT. The line parallel to the X axis was the cutoff reference line. The upper and lower edges of boxplot represent the upper and lower quartile and the short black line in boxplot represent the median.


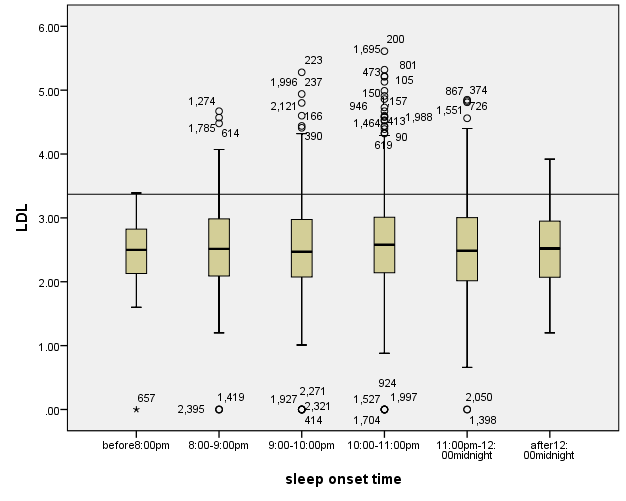
**Supplementary figure 3.** Distribution of TG among different SOT. The line parallel to the X axis was the cutoff reference line. The upper and lower edges of boxplot represent the upper and lower quartile and the short black line in boxplot represent the median.

| **Supplementary Table 1**. Regression model excludes participants with SOT bedtimes before 8:00pm or after 12:00 midnight (n=2,369) | | | | | |
| --- | --- | --- | --- | --- | --- |
|  | TG  *β* (95%*CI*) | TC  *β* (95%*CI*) | HDL  *β* (95%*CI*) | LDL  *β* (95%*CI*) | FPG#  *β* (95%*CI*) |
| Model 1 | 0.10(0.03,0.16)** | -0.31(-4.51,3.88) | -0.04(-0.06,-0.03)** | 0.05(0.01,0.08)* | -0.01(-0.03,0.01) |
| Model 2 | 0.08(0.02,0.15)* | -0.08(-4.35,4.19) | -0.04(-0.06,-0.02)** | 0.04(0.004,0.07)* | -0.01(-0.03,0.01) |
| Model 3 | 0.07(0.01,0.13)* | -0.12(-4.40,4.15) | -0.04(-0.05,-0.02)** | 0.03(0.0002,0.07)* | -0.005(-0.02,0.01) |
| Model 4 | 0.06(-0.02,0.14) | 2.19(-3.26,7.65) | -0.04(-0.06,-0.02)** | 0.04(0.01,0.09)* | -0.01(-0.03,0.02) |
| Model 1: sleep onset time + demographic characteristics (age, gender, ethnicity, education, marital status, occupation, economic condition).  Model 2: Model 1+health-related behaviors (smoking, alcohol use, tea drinking, exercise).  Model 3: Model 2+physical health (diabetes mellitus, hypertension, BMI).  Model 4: Model 3 + sleep duration.  # The model does not include diabetes mellitus.  **:*P*<0.01 **p*<0.05 *β*: beta. | | | | | |

| **Supplementary Table 2** Regression models between sleep onset time and cardiometabolic biomarkers stratified by sex | | | | | |
| --- | --- | --- | --- | --- | --- |
| Female (n=1,523) | | | | | |
|  | Triglycerides  *β* (95%*CI*) | Total cholesterol  *β* (95%*CI*) | HDL  *β* (95%*CI*) | LDL  *β* (95%*CI*) | FPG#  *β* (95%*CI*) |
| Model 1 | 0.07(-0.01,0.14) | 0.04(-0.01,0.08) | -0.05(-0.07,-0.03)** | 0.02(-0.02,0.06) | -0.01(-0.03,0.02) |
| Model 2 | 0.06(-0.01,0.14) | 0.55(-5.91,7.02) | -0.06(-0.08,-0.04)** | 0.02(-0.04,0.06) | -0.01(-0.03,0.01) |
| Model 3 | 0.07(-0.001,0.14) | 0.03(-0.01,0.08) | -0.04(-0.06,-0.03)** | 0.02(-0.04,0.07) | -0.01(-0.03,0.02) |
|  |  | Male (n=895) | | |  |
|  | Triglycerides  *β* (95%*CI*) | Total cholesterol  *β* (95%*CI*) | HDL  *β* (95%*CI*) | LDL  *β* (95%*CI*) | FPG#  *β* (95%*CI*) |
| Model 1 | 0.15(0.06,0.25) ** | -2.14(-12.03,7.74) | -0.04(-0.06,-0.02)** | 0.09(0.04,0.14) ** | -0.02(-0.06,0.01) |
| Model 2 | 0.14(0.04,0.24) ** | -2.72 (-12.76,7.32) | -0.04(-0.06,-0.02) ** | 0.09(0.04,0.14) ** | -0.03(-0.06,0.03) |
| Model 3 | 0.16(0.06,0.26) ** | -02.81 (-12.97,7.34) | -0.04(-0.06,-0.02)** | 0.08(0.03,0.13) ** | -0.02(-0.05,0.01) |
| Model 1: sleep onset time + demographic characteristics (age, gender, ethnicity, education, marital status, occupation, economic condition).  Model 2: Model 1+health-related behaviors (smoking, alcohol use, tea drinking, physical exercise).  Model 3: Model 2+physical health (diabetes mellitus, hypertension, BMI).  # The model does not include diabetes mellitus.  ** *p* <0.01 **p*<0.05 *β*: beta; *95%CI*: 95% confident interval.  HDL: High-density lipoprotein cholesterol; LDL: low-density lipoprotein cholesterol; FPG: fasting plasma glucose.  All the β are for sleep onset time. | | | | | |
